# Contemporary HCV pangenotypic DAA treatment protocols are exclusionary to real world HIV-HCV co-infected patients

**DOI:** 10.1186/s12879-019-3974-7

**Published:** 2019-05-03

**Authors:** A. Maughan, K. Sadigh, V. Angulo-Diaz, C. Mandimika, M. Villanueva, J. K. Lim, O. Ogbuagu

**Affiliations:** 1 Yale AIDS Program, Section of Infectious Diseases, Yale University School of Medicine, 135 College Street, Suite 323, New Haven, CT 06510 USA; 2 Department of Medicine, Yale-New Haven Hospital, New Haven, CT USA; 3 Section of Digestive Diseases, Department of Medicine, Yale University School of Medicine, New Haven, CT USA

**Keywords:** HIV, Hepatitis C, Direct-acting antivirals, Sofosbuvir, Velpatasvir, Glecaprevir, Pibrentasvir

## Abstract

**Background:**

Treatments for Hepatitis C virus (HCV) infection have vastly improved over the past few decades with current regimens now offering pangenotypic activity with excellent cure rates reported in clinical trials, including in the HIV-HCV coinfected population. However, there is some concern that stringent inclusion and exclusion criteria in the trials may lead to results that are not achievable in real-world populations.

**Methods:**

Our study evaluated a real-world HIV-HCV coinfected population and compared them to the eligibility criteria for trials of two of the most recent approved HCV agents; sofosbuvir/velpatasvir and glecaprevir/pibrentasvir.

**Results:**

Our study included 219 HIV-HCV coinfected patients and found that 89% met exclusion criteria for the sofosbuvir/velpatasvir trial and 90% met exclusion criteria for the glecaprevir/pibrentasvir trial. The majority of patients met more than one exclusion criteria with the most frequent criteria for exclusion being a non-approved ART regimen (58 and 47% respectively), having a psychiatric disorder (52%), active alcohol or injection drug use (27%), having an HIV viral load > 50 copies/ml (15%), a CrCl < 60 ml/min (13%) and a history of decompensated cirrhosis (13%).

**Conclusion:**

Although the newer Hepatitis C treatments are very effective, the real world HIV-HCV coinfected population often have comorbidities and other characteristics that make them ineligible for clinical trials, such that they are barriers to treatment. These barriers need to be recognized and addressed in order to optimize treatment outcomes in the HIV patient population.

## Background

In the recent decade, antiviral treatments for HCV have improved dramatically with treatment outcomes that were previously thought to be unachievable. Direct acting antivirals (DAA) therapy, unlike precursor therapies, has demonstrated very high cure rates among patients with chronic HCV infection regardless of previously recognized negative predictors of positive treatment response including high viral load, HCV genotype, and HIV infection status [1]. The next step in the public health response to the HCV epidemic, following the availability of these treatments, is to increase the number of people who both access and utilize these treatments and are cured.

However, the treatment cascade for HCV infection in the US shows that there are significant barriers to achieving treatment goals including suboptimal identification of individuals who are chronically infected, poor linkage to care where identified and low treatment and cure rates [2]. Some of the barriers for treatment include concerns about cost of treatment, provider availability and willingness to treat HCV-infected persons, patient level factors such as homelessness and substance use that may impact medication adherence as well as drug interactions which preclude the use of certain DAA regimens. These factors disproportionately impact individuals living with HIV infections [3, 4].

While recent clinical trials have shown robust cure rates, their applicability to real world populations remains in question. The necessity of imposing rigid eligibility criteria for clinical trial may limit generalizability of the results. Historically, persons living with HIV (PLWH) were either excluded or underrepresented in earlier HCV DAA trials but more contemporary ones have had trials dedicated to that cohort. However, some studies suggest that the entry criteria for such studies for HIV-HCV infected patients are so restrictive that relatively few individuals are eligible (including the requirement for virologic control of HIV infection and restrictions on antiretroviral drugs that have drug interaction potential) [5].

This is important because, of the estimated 4 million individuals with chronic HCV infection in the US, about 400,000 are HIV co-infected [6]. Conversely, among PLWH, up to 40% have HCV infection [6, 7]. Therefore expanding treatment to this population is of paramount public health importance.

This study sought to identify real-world eligibility of HIV-HCV co-infected individuals for contemporary DAA regimens and to identify modifiable and non-modifiable characteristics that impede eligibility for DAA therapy.

## Methods

### Study design/ setting/ participants

This was a cross sectional study of HIV–HCV co-infected patients at the Yale-New Haven Health system HIV clinic (Nathan Smith clinic). The Yale-New Haven HIV clinic is the largest HIV clinic in the state of Connecticut, with over 20 physicians and mid-level providers, who provide specialty care and services to over 1500 patients primarily residing in the Greater New Haven area majority of whom are male (60%) and racial or ethnic minorities (60%). Consultation and follow-up management for HCV therapy in the HIV clinic is typically performed by 2–3 physician providers once a week. Study data was collected between January 1, 2016 and December 31, 2016 and reflects the time point at which patient records were reviewed.

### Definitions and patient characteristics

HIV infection was defined as patients who had a positive Ab test, Ag/Ab test, and or positive HIV RNA assay historically (lower limit of detection [LLOD] of the lab assay was < 20 copies/ml). HCV infection was defined as a positive HCV antibody test and/or at least one detectable HCV RNA assay (HCV assay detected > 15 IU/ml). HIV and HCV viral load testing was performed using COBAS Ampliprep/COBAS Taqman, HIV and HCV version 2.0, Pleasanton California, USA respectively. HCV genotyping was performed using Versant HCV genotype, 2.0 assay (LiPA), Siemens Healthineers, Erlagen, Germany. Active substance use was defined as any drug use in the past 12 months. Cirrhosis status was defined as per liver biopsy, results of a single non-invasive test algorithm interpreted with recommended cut-offs for each test (APRI [score > 1], Fib-4 [score > 3.25], or fibrospect [score > 17] are used at our facility) or by ICD-9/10 codes in chart. Decompensated cirrhosis was defined as the presence of ascites, history of portosystemic encephalopathy, and/or variceal bleeding. Prior treatment for HCV was captured regardless of agent used, treatment duration or outcome. For patients on cART, the regimen captured was the regimen that the patient was on at the time of data collection. Mental health (psychiatric) disorders were captured by diagnostic codes (DSM 5) based on chart documentation by providers. Other patient characteristics, comorbidities and laboratory values were captured and reflect the most recent values at the time of chart review for all patients.

### Eligibility criteria

Patients were included in the analysis if they were (1) adults age > 18 years, (2) HIV infected (3) had laboratory evidence of HCV infection (as described previously). Among all patients who were registered at the YNHH HIV clinic, HIV-HCV coinfected patients were identified through an electronic medical records search utilizing the YNHH joint data analytics team (J-DAT team), where cases were identified based on a series of diagnostic codes and/or available laboratory values for both HIV and HCV infection.

### HCV clinical trial eligibility assessment

Study protocol eligibility criteria of the following pangenotypic HCV-DAA trials which were either inclusive of or exclusively for HIV-HCV coinfected patients were matched to characteristics of identified eligible patients: sofosbuvir-velpatasvir (SOF-VEL) ASTRAL-1 [8], glecaprevir-pibrentasvir (GLE-PIB) EXPEDITION-2 [9]. These are the first 2 pan-genotypic regimens approved for the treatment of chronic HCV infection in the United States. Complete inclusion and exclusion criteria were extracted from the study protocols (obtained from published manuscripts and from clinicaltrials.gov website) and utilized to create a database grid to which the criteria were matched to patient characteristics and data including labs.

### Data analysis/ statistics

Frequency tables were created to assess the proportion of enrolled patients who met all inclusion criteria AND at least one individual exclusion criteria. Simple frequencies were reported for each exclusion criteria as well as the total number of enrolled patients who met one or more of the exclusion criteria. Subgroup analyses were performed to assess for differences in characteristics and proportion of patients meeting each eligibility criteria for patients who were black or non-black as well as male versus female (latter assessed for differences in overall exclusion rates) utilizing N-1 Chi square test or Fishers exact test with significance set at a *P* value of 0.05.

## Results

### Patient demographic profiles

A total of 219 patients with HIV/HCV co-infection were included in our study. Our study population was predominantly male (67%), with 53% being of black race, 31% white and 14% of Hispanic ethnicity. The median age was 56 years with a range of 28–74 years [Table 1].

**Table 1:** Clinical characteristics and Demographics of HIV-HCV coinfected patient population at Yale-New Haven Hospital HIV Clinic

Characteristic	Total patients *N* = 219 (% or range)	Characteristic	Total patients *N* = 219 (% or range)
Age in years, median (range)	56 (28–74)	HIV viral load < 20 copies/mL, no. (%)	174 (80)
Race / ethnicity, no. (%)		CD4 count, median (range)	513 (27–2060)
Black	117 (53)	On ART, no. (%)	205 (94)
White	68 (31)	NRTI backbone	
Hispanic	30 (14)	Tenofovir (TDF or TAF)/ emtricitabine	135 (62)
Other	4 (2)	Abacavir/ lamivudine	47 (22)
Gender, no. (%)		NNRTI	
Male	147 (67)	Efavirenz	36 (16)
Female	72 (33)	Rilpivirine	22 (10)
HCV Genotype, no. (%)		Etravirine	10 (4.6)
1a	107 (49)	Protease inhibitors/ ritonavir	
1b	27 (12)	Atazanavir	30 (14)
2	5 (2)	Darunavir	31 (14)
3	6 (3)	Lopinavir	4 (2)
4	5 (2)	Integrase inhibitors	
Unknown	69 (32)	Dolutegravir	51 (23)
HCV viral load, mean IU/mL (range)	5.83 M (15–69,000,000)	Raltegravir	46 (21)
Cirrhosis status, no. (%)		Elvitegravir	10 (5)
Non-cirrhotic	79 (36)	Comorbidities, no (%)	
Compensated	111 (51)	EtOH use	184 (84)
Decompensated	29 (13)	Current	51 (23)
HCV prior treatment, no. (%)		Prior	133 (61)
Yes	54 (25)	IVDU	192 (88)
No	156 (71)	Current	32 (15)
Unknown	9 (4)	Prior	160 (73)
HBV Sag positive, no. (%)	0 (0)	Psychiatric disorder	114 (52)
		*Abnormal Creatinine Clearance	40 (18)

*ART* antiretroviral therapy, *HBV*, *EtOH* ethanol (alcohol), *Hepatitis B virus*; *HCV* Hepatitis C virus, *HIV* Human immunodeficiency virus, *IVDU* intravenous drug use, *NNRTI* non-nucleoside(tide) reverse transcriptase inhibitor, *TAF* tenofovir alafenamide, *TDF* tenofovir disoproxil fumarate

*Abnormal creatinine clearance < 60ml/min

### HIV and HCV characteristics

The majority of our patients had HCV genotype 1 (61%) with 49% having 1a and 12% with 1b; 32% had no genotypes recorded. Among the cohort, 64% had liver cirrhosis, 21% of whom had a history of decompensation. One-quarter (25%) had received prior treatment for their HCV infection. No patients had a positive Hepatitis B surface antigen test. Regarding HIV status, 94% of patients were on ART (49% being on integrase strand transfer inhibitor [INSTI] based regimens) with 80% having an HIV viral load < 20 copies/ml. Specific ART medications are shown in Table 1.

### Medical comorbidities

An overwhelming majority (88%) of patients had a history of intravenous drug use with 15% actively injecting, 84% with a history of alcohol use. Over half (52%) of the patients also had a DSM-5 psychiatric diagnosis such as depression, bipolar disorder, anxiety or schizophrenia. About 18% had chronic kidney disease (CKD) stage 3 or greater.

### Clinical trial eligibility – SOF/VEL

When eligibility criteria from the sofosbuvir/velpatasvir ASTRAL-1 trial was applied to our cohort, 89% of met at least one exclusion criteria with the most common reason for exclusion being on a non-approved antiretroviral regimen. When the antiretroviral regimen was removed as an exclusion criterion, 76% of patients remained ineligible. Other major exclusion criteria met by a significant number of our cohort included having a psychiatric disorder (52%), active alcohol or injection drug use (27%), having an HIV viral load > 50 copies/ml (15%), a CrCl < 60 ml/min (13%) and a history of decompensated cirrhosis (13%) [Table 2].

**Table 2:** Selective exclusion criteria and number of patients excluded

Exclusion criteria	Trial specific criteria	SOF/VEL ASTRAL-1 *N* = 219, no. (%)	GLE/PIB Expedition-2 *N* = 219, no. (%)
HIV VL	> 50 copies/mL	32 (15)	
	> 20 copies/ mL		44 (20)
ART regimen		127 (58)	103 (47)
CD4 count	< 100 cells/mm^3^	9 (4)	
	< 200 cells/mm^3^		28 (13)
Hepatic decompensation		29 (13)	29 (13)
Other liver disease (HBV, NASH, hemochromatosis)		5 (2)	5 (2)
Solid organ transplantation		2 (1)	2 (1)
Psychiatric disorder		114 (52)	114 (52)
Malignancy (within previous 5 yrs)		12 (5)	12 (5)
Active EtOH or IVDU		59 (27)	59 (27)
ALT > 10x ULN		1 (< 1)	1 (< 1)
AST > 10x ULN		2 (1)	2 (1)
D. bili > 3 mg/dL		8 (4)	8 (4)
Platelets	< 50,000/μL	11 (5)	
	< 60,000/μL w/ cirrhosis or < 90,000/μL w/o cirrhosis		22 (10)
HbA1c > 8.5%		5 (2)	5 (2)
CrCl	< 60 mL/min	28 (13)	
	< 50 mL/min		20 (9)
Hemoglobin	< 10 g/dL	16 (7)	
	< 12 g/dL (men), < 11 g/dL (women)		39 (18)
Albumin < 3 g/dL		19 (9)	19 (9)
INR	> 1.5x ULN	6 (3)	
	> 2.3		6 (3)
Overall excluded		195 (89)	198 (90)
Overall excluded without ART regimen criterion		167 (76)	178 (81)

*ALT* alanine aminotransferase, *ART* antiretroviral therapy, *AST* aspartate aminotransferase, *bili* bilirubin, *CrCl* creatinine clearance, *EtOH* ethanol (alcohol), *GLE* glecaprevir, *HBV* Hepatitis B virus, *HCV* Hepatitis C virus, *HIV* Human immunodeficiency virus, *INR* international normalized ratio, *IVDU* intravenous drug use, *NASH* non-alcoholic steatohepatitis, *PIB* pibrentasvir, *SOF* sofosbuvir, *VEL* velpatasvir, *VL* viral load

Protocols for SOF/VEL ASTRAL-1 and GLE/PIB EXPEDITION-2 studies may be obtained from:

https://www.nejm.org/doi/suppl/10.1056/NEJMoa1512610/suppl_file/nejmoa1512610_protocol.pdf (SOF/VEL) and https://www.ncbi.nlm.nih.gov/pmc/articles/PMC6137115/ (GLE/PIB)

### Clinical trial eligibility – GLE/PIB

For the glecaprevir/pibrentasvir Expedition-2 trial, 90% of patients met at least one exclusion criteria and 81% remained ineligible if the antiretroviral regimen was not included as an exclusion criterion. Top exclusion criteria met included: having a psychiatric disorder (52%), a non-approved ART regimen (47%), active alcohol or IDU (27%), having a viral load > 20 copies/ml (20%) and hemoglobin < 12 g/dl for men and < 11 g/dl for women (18%), a CD4 count < 200 cells/mm3 (13%) and a history of decompensated liver cirrhosis (13%) [Table 2].

### Subgroup analyses

There were no statistically significant differences in overall proportion of excluded patients when comparing patients by gender or ethnicity [Table 3]. However, when proportions of patients meeting specific exclusion criteria were compared between black versus non-black patients, there was a significant difference for those with active alcohol or IDU (38% versus 14%, *P* < 0.001) [Table 4].

**Table 3:** Subgroup analysis (proportion of patients meeting exclusion criteria by gender and ethnicity)

Characteristic	SOF/VEL ASTRAL-1 No. excluded (%)	Difference (sig level)	GLE/PIB Expedition-2 No. excluded (%)	Difference (sig level)
Gender				
Female (*N* = 72)	65 (90)		67 (93)	
Male (*N* = 147)	130 (88)		131 (89)	
		1.9% (p 0.673)		3.9% (p 0.359)
Ethnicity				
Black (*N* = 117)	108 (92)		110 (94)	
Non-black (*N* = 102)	87 (85)		88 (86)	
		7% (p 0.099)		7.7% (p 0.054)

*GLE* glecaprevir, *PIB* pibrentasvir, *SOF* sofosbuvir, *VEL* velpatasvir

**Table 4:** Selective exclusion criteria and number of patients excluded by race (black versus non-black)

Exclusion criteria	Trial specific criteria	SOF/VEL ASTRAL-1 no. (%)		Difference (sig level)	GLE/PIB Expedition-2 no. (%)		Difference (sig level)
		Black *N* = 117	Non-black *N* = 102		Black *N* = 117	Non-black *N* = 102	
HIV VL	> 50 copies/mL	16 (14)	16 (16)	2% (*p* 0.677)			
	> 20 copies/ mL				22 (19)	22 (22)	2.8% (*p* 0.607)
ART regimen		70 (60)	57 (56)	3.9% (*p* 0.561)	60 (51)	43 (42)	9% (*p* 0.184)
CD4 count	< 100 cells/mm^3^	5 (4)	4 (4)	0.4% (*p* 0.585)			
	< 200 cells/mm^3^				15 (13)	13 (13)	0.1% (*p* 0.982)
Hepatic decompensation		13 (11)	16 (16)	4.6% (*p* 0.318)	13 (11)	16 (16)	4.6% (*p* 0.318)
Other liver disease (HBV, NASH, hemochromatosis)		**0**	**5 (5)**	**4.9%** (**p 0.021**)	**0**	**5 (5)**	**4.9% (** ***p*** **0.021)**
Solid organ transplantation		1 (< 1)	1 (< 1)	0.13% (*p* 0.716)	1 (< 1)	1 (< 1)	0.13% (*p* 0.716)
Psychiatric disorder		58 (50)	56 (55)	5.3% (*p* 0.435)	58 (50)	56 (55)	5.3% (*p* 0.435)
Malignancy (within previous 5 yrs)		**10 (8)**	**2 (2)**	**6.5% (** ***p*** **0.039)**	**10 (8)**	**2 (2)**	**6.5% (** ***p*** **0.039)**
Active EtOH or IVDU		**45 (38)**	**14 (14)**	**24.8% **(***p*** **< 0.001**)	**45 (38)**	**14 (14)**	**24.8% (*p*** **< 0.001)**
ALT > 10x ULN		0	1 (< 1)	0.98% (*p* 0.466)	0	1 (< 1)	0.98% (*p* 0.466)
AST >10x ULN		1 (< 1)	1 (< 1)	0.13% (*p* 0.716)	1 (1)	1 (1)	0.13% (*p* 0.716)
D. bili > 3 mg/dL		4 (3)	4 (4)	0.5% (*p* 0.561)	4 (3)	4 (4)	0.5% (*p* 0.561)
Platelets	< 50,000/μL	7 (6)	4 (4)	2.1% (*p* 0.549)			
	< 60,000/μL w/ cirrhosis or < 90,000/μL w/o cirrhosis				11 (9)	11 (11)	1.4% (*p* 0.732)
HbA1c > 8.5%		5 (4)	0	4.3% (*p* 0.063)	5 (4)	0	4.3% (*p* 0.063)
CrCl	< 60 mL/min	18 (15)	10 (10)	5.6% (*p* 0.217)			
	< 50 mL/min				14 (12)	6 (6)	6.1% (*p* 0.119)
Hemoglobin	< 10 g/dL	10 (8)	6 (6)	2.6% (*p* 0.450)			
	< 12 g/dL (men), < 11 g/dL (women)				25 (21)	14 (14)	7.7% (*p* 0.138)
Albumin < 3 g/dL		11 (9)	8 (8)	1.6% (*p*0.675)	11 (9)	8 (8)	1.6% (*p* 0.675)
INR	>1.5x ULN	3 (3)	3 (3)	0.3% (*p* 0.592)			
	> 2.3				3 (3)	3 (3)	0.3% (*p* 0.592)
Overall excluded		108 (92)	87 (85)	7% (*p* 0.099)	110 (94)	88 (86)	7.7% (*p* 0.054)
Overall excluded without ART regimen criterion		92 (79)	75 (74)	5.1% (*p* 0.378)	100 (85)	78 (76)	9% (*p* 0.089)

*ALT* alanine aminotransferase, *ART* antiretroviral therapy, *AST* aspartate aminotransferase, *bili* bilirubin, *CrCl* creatinine clearance, *EtOH* ethanol (alcohol), *GLE* glecaprevir, *HBV* Hepatitis B virus, *HCV* Hepatitis C virus, *HIV* Human immunodeficiency virus, *INR* international normalized ratio, *IVDU* intravenous drug use, *NASH* non-alcoholic steatohepatitis, *PIB* pibrentasvir, *SOF* sofosbuvir, *VEL* velpatasvir, *VL* viral load

## Discussion

The cascade of HCV care in the United States has been shown to be suboptimal with very few individuals with chronic infection who have achieved a cure [10]. While there are many contributors to this deficit [11], efforts have to be targeted to better understanding and addressing barriers to treatment where identified.

Multiple studies have shown that HIV infected individuals achieve similar HCV treatment results compared to their uninfected counterparts [12–14]. However, it is recognized that while efficacy of novel DAA therapies have resulted in excellent cure rates, the studies typically enroll optimal patient populations (including HIV infected patients) with stringent eligibility criteria; however the real world effectiveness is lower suggesting that many patients may not be eligible for using the therapies [5]. This was noted strikingly in our study results where out of the 219 HIV infected patients who were reviewed, approximately 90% met at least 1 exclusion criteria. While drug-drug interactions with current ART was a primary cause of ineligibility [see Table 5], even when the antiretroviral regimen exclusion criteria were removed, a significant proportion of patients remained ineligible for study inclusion. This has significant implications in the real world as it suggests that even if all patients were able to be transitioned to an approved ART regimen, the majority of patients would have still been excluded due to other co-morbidities or laboratory abnormalities, the most common of which were psychiatric illness, active drug and alcohol use and having a detectable HIV viral load.

**Table 5:** Drug interactions of HCV Direct Acting Antiviral agents with ART^a^

HCV DAA	ART drug name	Effect on concentration	Comments
Sofosbuvir/Velpatasvir			
	Efavirenz	↓ velpatasvir	Coadministration not recommended
	Tenofovir disoproxil (TDF)	↑ tenofovir	Renal monitoring for tenofovir associated adverse reactions.
	Tipranavir/ritonavir	↓ sofosbuvir ↓ velpatasvir	Coadministration not recommended
Glecaprevir/Pibrentasvir			
	Atazanavir	↑ glecaprevir ↑ pibrentasvir	Coadministration contraindicated due to increased risk of ALT elevations
	Darunavir/Lopinivir/ritonovir	↑ glecaprevir ↑ pibrentasvir	Coadministration not recommended
	Efavirenz	↓ glecaprevir ↓ pibrentasvir	Coadministration not recommended

*DAA* direct acting antiviral, *HCV* hepatitis C virus, *ALT* alanine aminotransferase

^a^Reference: Hepatitis C guidance: AASLD-IDSA recommendations for testing, managing, and treating adults infected with hepatitis C virus. *Hepatology.* 2015;62(3):932–954

The high rates of drug-drug interactions (DDIs) between current cART and DAA therapy have been shown in other studies. A Dutch study which evaluated DDI among 418 HIV/HCV coinfected individuals who were on ART, found that 47% had a category 2/3 DDI between HCV DAA and their cART prior to initiation of DAA treatment. Regimen switches were carried out in 51% of those with a category 2 DDI, while 98% of patients with a category 3 DDI had a regimen switch [15]. Another study showed that 64–76% of HIV/HCV coinfected patients had to switch their ART to minimize DDI concerns with HCV DAA regimens [16]. However, given changing HIV treatment guidelines and a preference contemporarily for INSTI based therapies, DDI are likely to be less of a concern in the future, although in resource limited settings, this may not be the case. This might also be problematic in people with drug resistant HIV virus that require specific ARVs like PI based or containing regimens.

Another key eligibility criteria that was problematic for our cohort was the presence of mental health disorders. Part of the concern is that it is not clear which specific disorders and what severity preclude HCV treatment such that providers may make subjective and possibly inappropriate decisions to withhold HCV therapies in these patients. Clinical trials have shown that mental health disorders such as depression [17], and psychotic disorders [18] can severely limit an individual’s capacity to adhere to medical treatments. In these patient populations, interventions such as psychosocial intervention, cognitive based therapies, adherence-coping-education (ACE) cognitive adaptation training are some interventions that hold promise to improve adherence rates and could be incorporated utilizing a multidisciplinary care model which already exists in many HIV treatment care centers [18].

Regarding alcohol and other substance use, similar to mental health disorders, study protocols typically state that for individuals with active or recent use (within 6–12 months,) they are left to the discretion of investigators to determine their ability to adhere to study protocols. This typically leads to study exclusion. However, while active injection drug is frequently an exclusionary consideration in HCV trials, studies have shown that such individuals may be successfully treated with DAA therapy [19, 20]. This is particularly noteworthy as people who inject drugs (PWID) are disproportionately impacted by HCV infection (with prevalence rates up to 39%) such that HCV elimination efforts must include the population to be successfully achieved [21].

The requirement for HIV disease control in patients who are candidates for HCV therapy may also be inappropriate. While the current management paradigm of HIV disease is lifelong ART with disease control, HCV can be cured with relatively short term therapy (8–12 weeks) in almost all patients including those with HIV. Therefore, while poor adherence can cut across comorbid conditions in an individual, and poor control of one disease may be a reliable surrogate for risk of poor adherence to the treatment of another comorbidity, individuals may be motivated by a finite and short duration of therapy and the prospect of a cure to preferentially adhere to HCV therapy. Similarly, having a low CD4 count as an exclusion criteria may not be clinically appropriate.

Certain exclusion criteria may not be modifiable when identified such as presence of liver cirrhosis with decompensation. However, even in those individuals, referral to or consultation with an HCV expert may facilitate treatment.

Our cohort which had a majority of patients of Black race could have faced unique barriers to study eligibility. In our cohort, black individuals had significantly higher rates of active alcohol or injection drug use compared to non-black individuals. Typically, black patients are underrepresented in HCV clinical trials despite being disproportionately impacted by HCV [22]. Contributors to this decreased eligibility may reflect patient characteristics such as substance use or homelessness. However, there are suggestions in the literature that provider bias rather than these cited reasons may be an important factor in black patients being deemed ineligible for HCV therapy [23]. To overcome some of these barriers, a multidisciplinary treatment model with integrated care for substance use, mental health disorder, in many cases which already exist within HIV clinic frameworks, may be employed to optimize HCV therapy. There are already some examples of successful real world treatment of HIV/HCV coinfected individuals that demonstrate that stringent exclusion criteria in clinical trials do not necessarily need to be invoked [24, 25].

### Limitations

We had a small sample size, utilized data from a single center and had a high proportion of patients who were black, male and had decompensated liver cirrhosis which may not reflect characteristics of other HCV-HIV infected cohorts such that it may limit the generalizability of our findings. We used robust definitions of psychiatric disorders, and didn’t quantify severity of alcohol and drug use, which may have led to overestimation of ineligibility rates. Patient characteristics were determined as documented by providers which can be incomplete, biased or inaccurate.

## Conclusion

While the cure rates of people with HIV-HCV coinfection are impressive, more efforts are needed to address barriers to treatment eligibility as have been identified in this and other studies. Contemporary evidence suggests that historically hard-to-treat patients such as substance users can be successfully treated for hepatitis C such that study designs should be expanded to include such populations. In addition, while not all barriers identified are modifiable, a multidisciplinary care approach may also result in more optimal management outcomes for these patients.

## Abbreviations

ALT: Alanine aminotransferase
ART: Antiretroviral therapy
AST: Aspartate aminotransferase
Bili: Bilirubin
CrCl: Creatinine clearance
EtOH: Ethanol (alcohol)
GLE: Glecaprevir
HBV: Hepatitis B virus
HCV: Hepatitis C virus
HIV: Human immunodeficiency virus
INR: International normalized ratio
IVDU: Intravenous drug use
NASH: Non alcoholic steatohepatitis
NNRTI: Non nucleoside(tide) reverse transcriptase inhibitor
PIB: Pibrentasvir
SOF: Sofosbuvir
TAF: Tenofovir Alafenamide
TDF: Tenofovir disoproxil fumarate
VEL: Velpatasvir
VL: Viral load
